# Modified Autologous Biceps Acromioclavicular and Horizontal Neutralization (AutoBAHN) Technique Using a Free Biceps Tendon Autograft for Acromioclavicular Joint Stabilization

**DOI:** 10.1002/atn2.70353

**Published:** 2026-11-14

**Authors:** Tobias Gruber, Eduard Van Eecke, Lukas Lance, Tim Leschinger, Markus Scheibel

**Affiliations:** ^1^ Department of Shoulder and Elbow Surgery Schulthess Clinic Zurich Switzerland; ^2^ Department of Orthopaedic and Trauma Surgery, Faculty of Medicine and University Hospital Cologne University of Cologne Cologne Germany; ^3^ Department of Orthopaedic Surgery AZ Delta Roeselare Belgium; ^4^ Department of Shoulder and Elbow Surgery, Center for Musculoskeletal Surgery Charité‐Universitaetsmedizin Berlin Berlin Germany

## Abstract

We present a modification to the autologous biceps acromioclavicular stabilization and horizontal neutralization technique. In contrast to the original technique, we propose releasing the long head of the biceps tendon (LHB) at the origin and securing the proximal portion to the scapular neck in either an inlay or onlay fashion. Similar to the original technique, the distal portion of the LHB is routed through the clavicle and secured with an interference screw. Next, the LHB is passed over the acromioclavicular joint and anchored in the lateral acromion, providing vertical and horizontal stabilization. This modification aims to preserve the fundamental biomechanical principles and advantages of the original technique, using a local, strong, and autologous graft that avoids donor site morbidity and allograft‐related concerns. This technique expands the indication, offering a practical and versatile alternative for acromioclavicular joint reconstruction when preservation of the native LHB insertion is not feasible or desirable.

## Graphical abstract

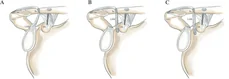

**VIDEO 1:** Modifications to the arthroscopically assisted autologous biceps acromioclavicular and horizontal neutralization technique using a free tendon graft are presented. The patient is placed in the beach‐chair position. After standard sterile prepping of the left shoulder, diagnostic glenohumeral arthroscopy through a standard posterior portal is performed. The long head of the biceps tendon is tenodesed and tenotomized near the musculotendinous junction using a subpectoral approach. Next, the origin of the long head of the biceps tendon is released through an anteroinferior working portal, and the free tendon graft is attached to the scapular neck in either an onlay or inlay manner. The distal portion of the long head of the biceps tendon is routed through the lateral clavicle and secured with an interference screw. Finally, the distal portion of the tendon is passed over the acromioclavicular joint and anchored to the lateral acromion. Standard wound closure is performed, and final radiographic images are obtained. Video content can be viewed at https://doi.org/10.1002/atn2.70353.

Management of symptomatic chronic acromioclavicular joint (ACJ) instability remains a topic of ongoing debate, most commonly combining synthetic fixation with biological graft augmentation.^1^, ^2^ Frequently used graft options present with specific limitations like limited biomechanical strength (coracoacromial ligament), availability, higher failure rates and costs (allografts), or donor site morbidity, including infection, hematoma formation, postoperative weakness, and muscle cramping (hamstring autografts).^3^, ^4^, ^5^, ^6^, ^7^ Recently, the autologous long head of the biceps tendon (LHB) has been discussed as a viable graft option.^8^, ^9^, ^10^, ^11^, ^12^

The autologous biceps acromioclavicular and horizontal neutralization (AutoBAHN) technique offers a local, robust, and cost‐efficient autograft option, avoiding additional donor site morbidity and preserving the native LHB origin (Figure 1A).^9^, ^13^ However, in the setting of concomitant intra‐articular lesions, preservation of the proximal LHB attachment is not always feasible. Reported arthroscopic prevalence of SLAP lesions ranges from approximately 3.9% to 26% of cases.^14^, ^15^, ^16^ Furthermore, anatomical variants of the origin (40%‐60%) and of the intra‐articular part of the LHB (1.9%‐7.4%) may not be suited for the original AutoBAHN technique.^17^, ^18^, ^19^ To address these clinical scenarios, we describe a modified AutoBAHN technique (Figure 1B and C), combining the advantages and biomechanical principles of the original technique with an onlay or inlay fixation of the proximal LHB at the scapular neck, thereby providing a reliable option when preservation of the native LHB origin may not be feasible or desirable.

**FIGURE 1:**
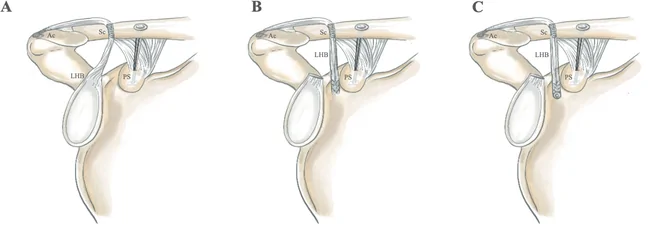
Schematic illustration of the AutoBAHN technique and its modifications, depicted from an anterolateral view. In the original technique (A), the LHB is rerouted through the clavicle, secured with an interference Sc and passed over the acromioclavicular joint and anchored in the lateral Ac. In addition, sutures from a PS are passed through the clavicle and coracoid base. Alternative fixation techniques include an onlay configuration (B) or an inlay configuration (C), in which the LHB is anchored at the scapular neck using either 2 anchors or a single anchor, respectively. (Ac, acromion; AutoBAHN, autologous biceps acromioclavicular and horizontal neutralization technique; LHB, long head of the biceps tendon; PS, pulley‐system device; Sc, screw.)

## SURGICAL TECHNIQUE

This procedure was developed in accordance with the Declaration of Helsinki. Written informed patient consent was obtained. The modified AutoBAHN technique for treating chronic symptomatic bidirectional ACJ instability involves arthroscopically assisted stabilization using a free autologous LHB graft augmented with a suture button pulley construct. The LHB is tenodesed and tenotomized near the musculotendinous junction. The origin of the LHB is released and secured in either an onlay or inlay fashion to the scapular neck with 1 or 2 anchors, respectively. The distal portion of the LHB is routed through the lateral clavicle and secured with an interference screw. Finally, the distal portion of the tendon is passed over the ACJ and anchored to the lateral acromion. Chronological surgical steps are outlined in Table 1. The surgical technique is shown in Video 1.

**TABLE 1:** Central Steps of the Modified AutoBAHN Technique

**Surgical Steps**
**1. Diagnostic arthroscopy**
Assess whether the use of the LHB is feasible
**2. Subpectoral LHB tenodesis and tenotomy**
Through a subpectoral approach, perform tenodesis and tenotomy near the musculotendinous junction
**3. LHB retraction and preparation**
Retrieve the tendon intra‐articularly and augment it with sutures in a baseball‐stitch manner
**4. Vertical ACJ stabilization**
Reduce and stabilize the ACJ with a pulley suture system
**5. Attachment of the proximal LHB**
Secure the proximal portion of the LHB to the scapular neck
**6. Transclavicular routing and fixation**
Route the distal portion of the LHB through the clavicle and secure it with an interference screw
**7. Horizontal ACJ stabilization**
Span the LHB over the ACJ and anchor it in the lateral acromion
**8. Wound closure and final imaging**
Close the clavicular incision in 2 layers, the others in conventional fashion and obtain final radiographic images

ACJ, acromioclavicular joint; AutoBAHN, autologous biceps acromioclavicular and horizontal neutralization technique; LHB, long head of the biceps tendon.

### Preoperative Planning

The diagnostic workup is identical to similar techniques, consisting of review of patient history, clinical examination, and comprehensive radiographic assessment.^9^

### Technique

#### Diagnostic Arthroscopy

The patient is positioned in the beach‐chair orientation, and standard sterile preparation, draping, and diagnostic glenohumeral arthroscopy through a posterior portal are completed. Special attention is given while assessing the SLAP complex and the integrity of the LHB.

#### Subpectoral LHB Tenodesis and Tenotomy

A mini‐open subpectoral approach is applied for LHB tenodesis and tenotomy. Tenodesis is performed close to the musculotendinous junction by tagging the LHB with a nonabsorbable suture (Force Fiber #2, Stryker, Mahwah, NJ) using a baseball stitch configuration. The tendon is then secured in an onlay manner using a 2.6‐mm unicortical biceps button (Arthrex, Naples, FL).

#### LHB Retraction and Preparation

Three additional arthroscopic portals are created: an anteroinferior working portal located above the subscapularis tendon, an anterolateral transtendinous viewing portal, and an anterolateral rotator interval “parking portal” for tendon management. The LHB is retrieved and retracted with a grasper through the “parking portal.” Next, the LHB is released at the origin at the supraglenoid tubercle, and the “free” LHB graft is extracted. There, the tendon is threaded with a nonabsorbable suture (Force Fiber #2, Stryker), and its diameter is measured using a sizing block in preparation for later passage from inferior, through the clavicle, to the superior aspect.

#### Vertical ACJ Stabilization

Vertical ACJ stabilization is achieved with a Low‐Profile TightRope (Arthrex) suture system^20^ (Figures 2A–Fand 3A–F).

**FIGURE 2:**
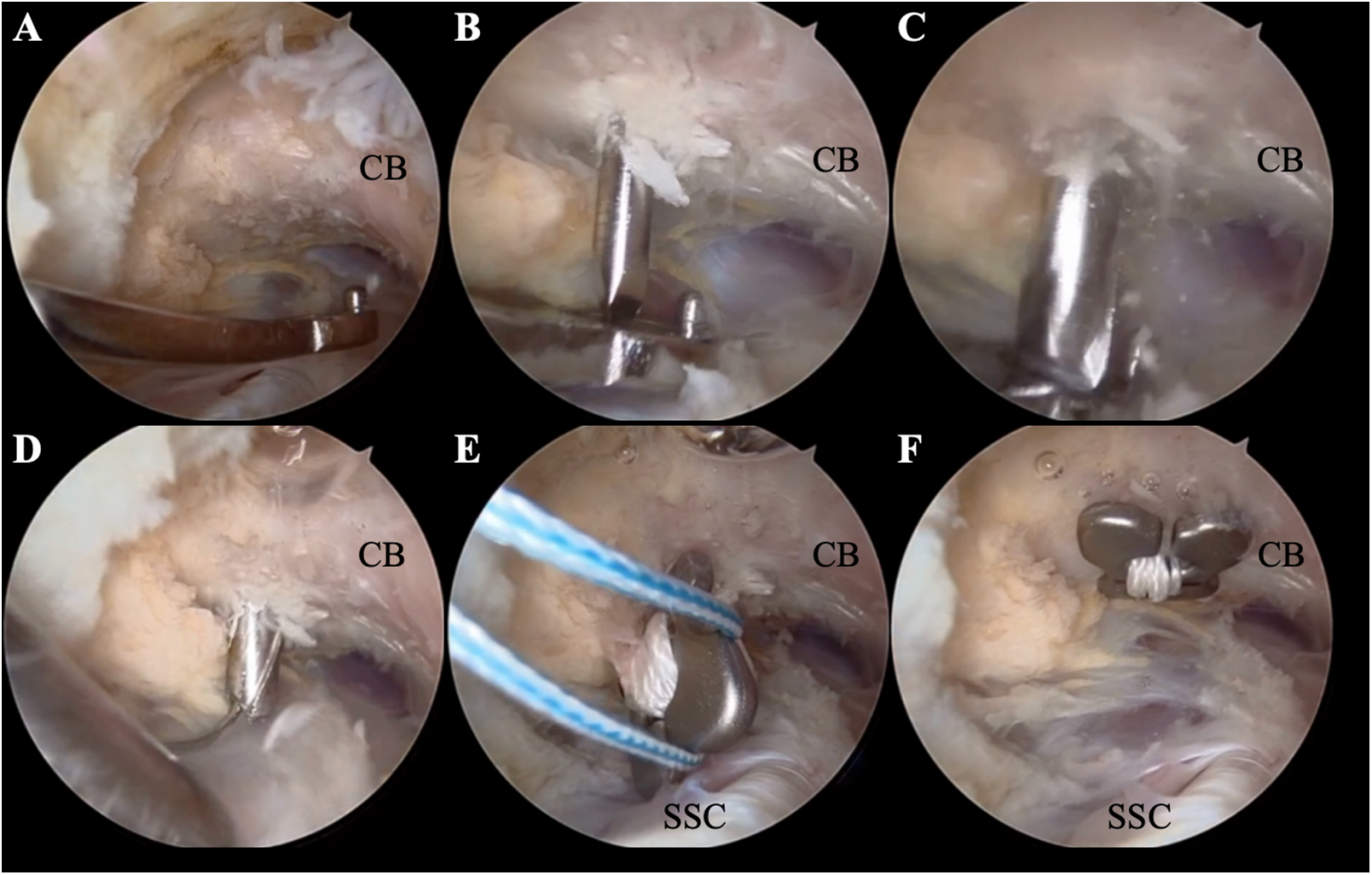
Arthroscopic images of vertical ACJ stabilization using a transtendinous viewing portal and an anteroinferior working portal. This is achieved with a Low‐Profile TightRope suture system (Arthrex, Naples, FL). The CB is exposed (A). A 2‐cm sagittal skin incision is made 3 cm medial to the ACJ over the superior clavicle. With the help of fluoroscopic imaging and an anterior cruciate ligament drill guide, a 2 mm K‐wire is advanced through the clavicle and coracoid (B). The K‐wire is overdrilled using a 3 mm cannulated drill (C), and at the superior clavicle a 5.1 mm unicortical socket is created. A nitinol suture passing wire is used for the shuttling of a FiberLink suture (Arthrex) (D). A Dog Bone button (Arthrex) is intertwined and introduced to the coracoid base (E) and ultimately fastened, and the ACJ is reduced from the superior clavicle using suture tensioners with 100 N of force (F). (ACJ, acromioclavicular joint; CB, coracoid base; SSC, subscapularis muscle.)

**FIGURE 3:**
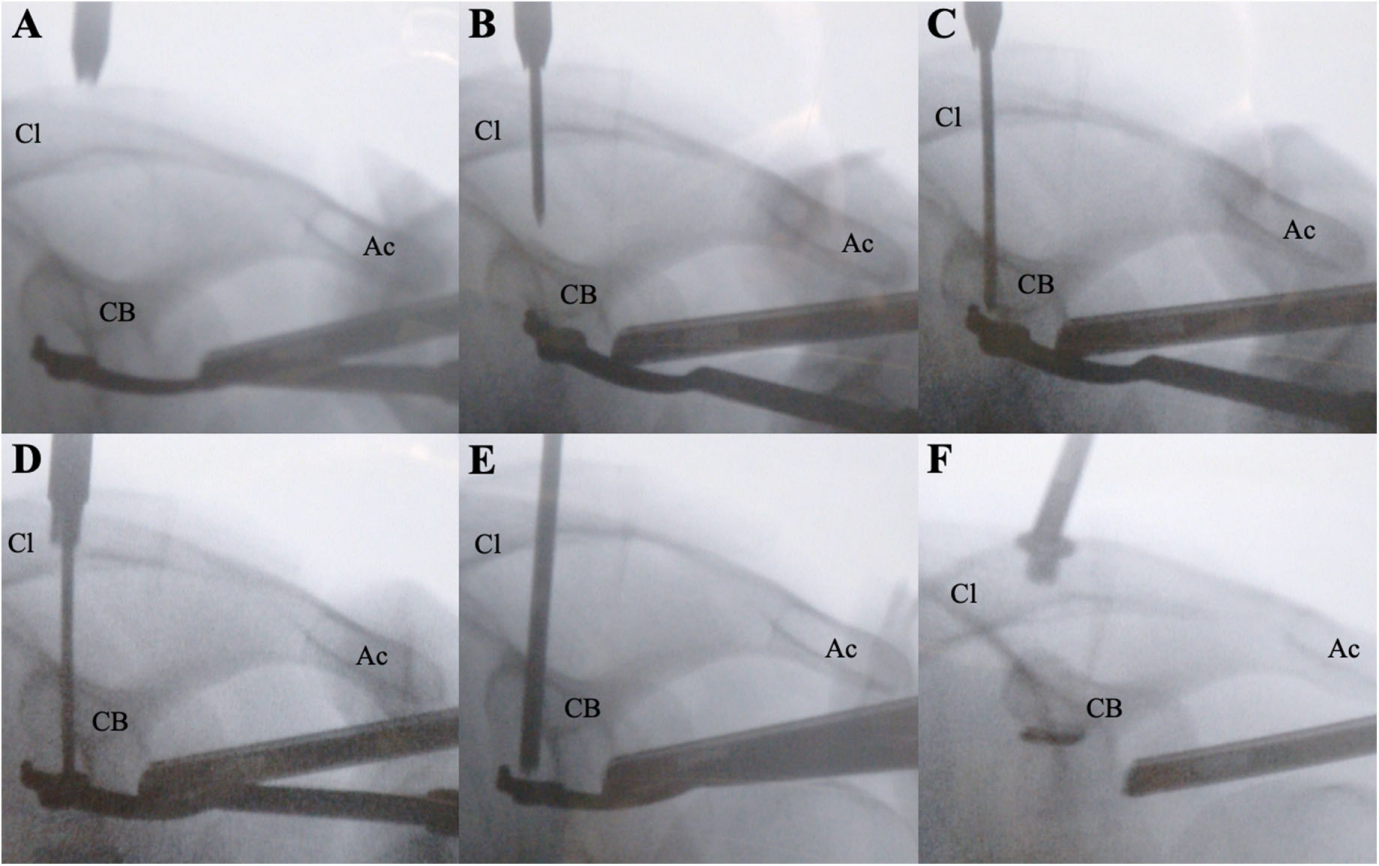
Radiographs of a left shoulder, viewed from anterior. Under radiological control and with the help of a drill guide, a K‐wire is passed through the clavicle and coracoid base (A–C). A 5.1‐mm unicortical socket is drilled at the superior clavicle, and a 3‐mm canal is drilled through the clavicle and coracoid base for implantation of a Low‐Profile suture system (D,E). Using suture tensioners and 100 N of force, the clavicle is reduced (F). (Ac, acromion; CB, coracoid base; Cl, clavicle.)

#### Attachment of the Proximal LHB

The proximal part of the LHB may be secured to the scapular neck either via an onlay or inlay fixation, with 1 or 2 anchors, respectively.

#### Onlay Fixation

The scapular neck is cleared from soft tissue and 2 single loaded all‐suture anchors are placed at the coracoid base (Figure 4A–C). One strand from each anchor is used to mark the proximal part of the free LHB graft in baseball stitch fashion. Similar to distal fixation of the short head of the biceps tendon, the other strands are passed through the tendon as a running suture to control reduction and tension of the free graft (Figure 4D).^21^ After adjusting the graft to the scapular, the suture limbs are knotted and released.

**FIGURE 4:**
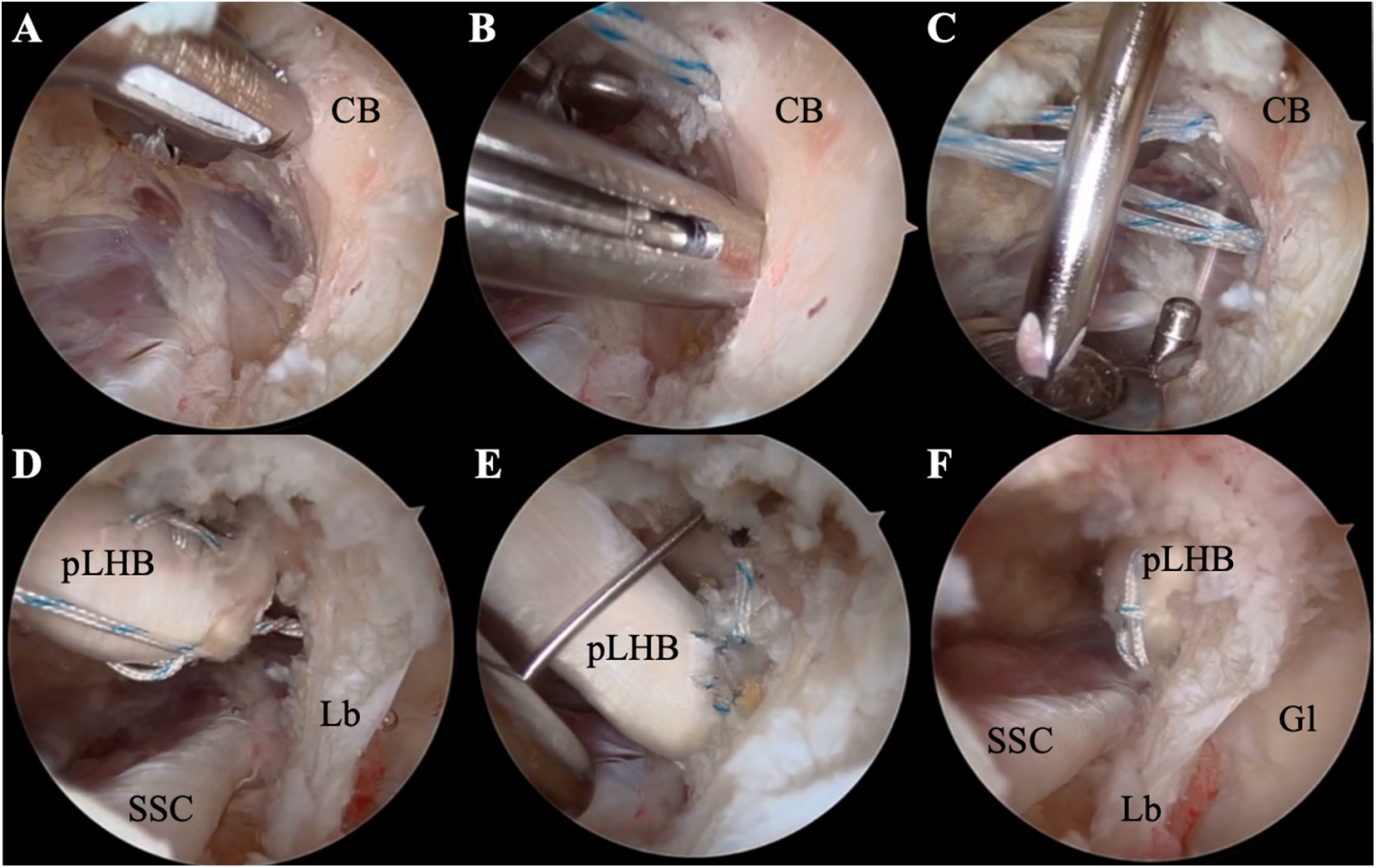
Arthroscopic images viewed from a transtendinous portal of an onlay fixation of the proximal pLHB at the CB. A 12° slant (Stryker, Mahwah, NJ) is inserted through the anteroinferior working portal, and an all‐suture anchor (Iconix 1.4 mm single loaded with #2 Force Fiber; Stryker) is placed at the coracoid base (A). A second identical anchor is placed inferior to the first (B). A second drill canal is created through the clavicle (C). Using the sutures of the anchors, the LHB is secured to the coracoid base (D). In the overview, the SSC and the Lb are depicted (D). Next, a nitinol suture passing wire is retrieved through the created canal (E). Finally, the LHB graft is routed through the clavicle (F). Adjacent to the attached graft, the SSC, the Lb, and the Gl are visible. (CB, coracoid base; Gl, glenoid; Lb, glenoid labrum; pLHB, portion of the long head of the biceps; SSC, subscapularis muscle.)

#### Alternative Inlay Fixation

Fixation of a free tendon graft may also be achieved using an inlay technique. Next to the coracoid base, a drill hole can be created, and the tendon can be blocked within the socket using a singular anchor. An arthroscopic grasper or additional sutures can be used to aid the intra‐articular adjustment of the tendon graft.

#### Transclavicular Routing and Fixation

To shuttle the LHB, the distal sutures of the tendon are retrieved anteroinferiorly and introduced into the eyelet of the transclavicular nitinol suture‐passing wire, routing the LHB through the clavicle (Figure 4E and F). Therefore, a second clavicular tunnel is created with a diameter of 5 mm (Figure 5A–D). The sutures are held under manual tension, whereas the tendon is secured within the clavicular tunnel using a 5.5 × 10 mm interference screw (Arthrex).

**FIGURE 5:**
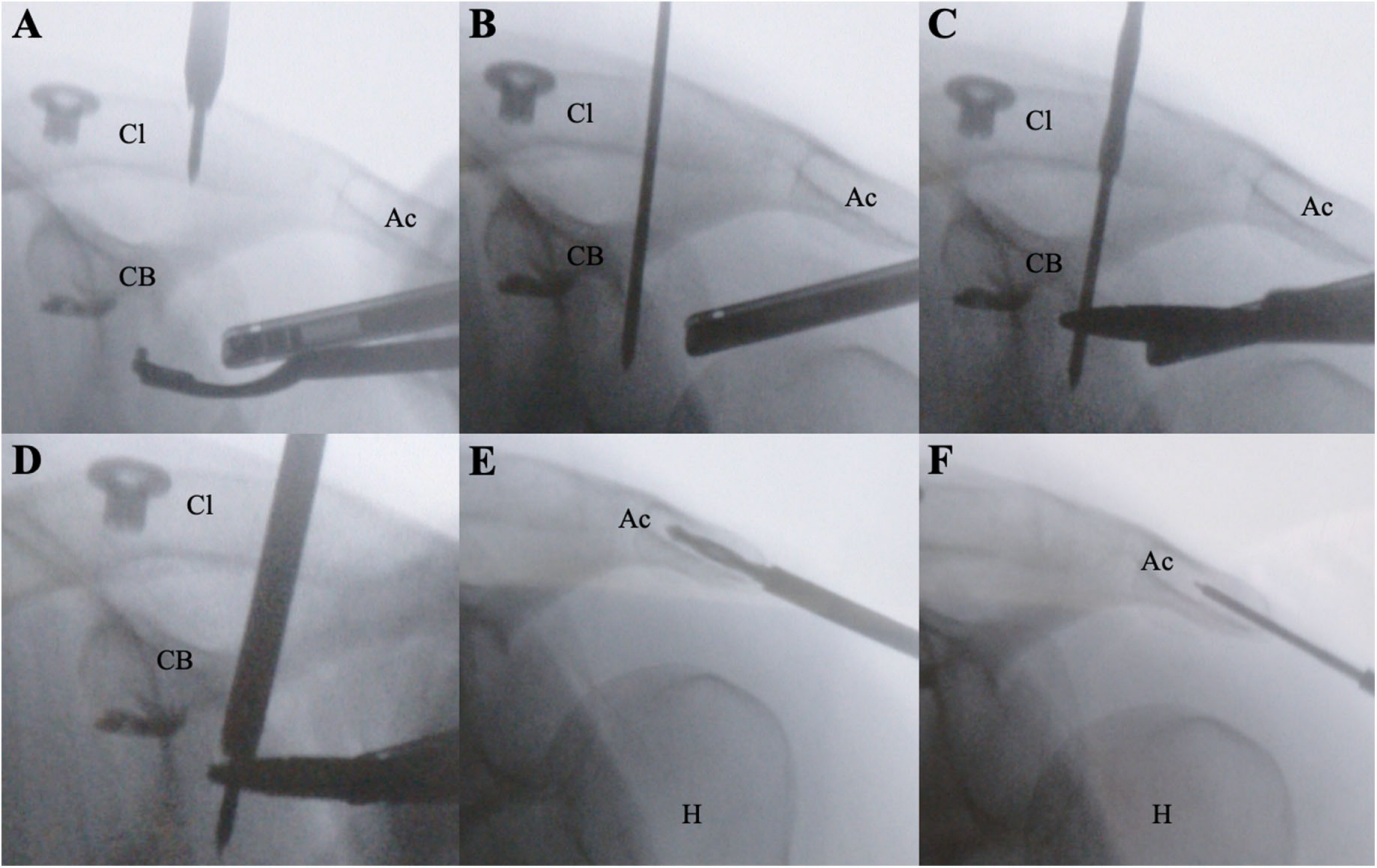
Radiographs of a left shoulder, viewed from anterior. An additional drill hole at the lateral clavicle is created using a K‐wire (A), without traversing the coracoid base (B). Subsequently, the K‐wire is overdrilled incrementally (C,D) until a 5 mm diameter is achieved. Once the tendon is routed through this canal, the LHB is secured with an interference screw within the clavicle, and the distal portion is passed over the acromioclavicular joint and anchored in a previously created drill hole at the middle part of the lateral acromion (E,F). (Ac, acromion; CB, coracoid base; Cl, clavicle; H, humeral head; LHB, long head of the biceps tendon.)

#### Horizontal ACJ Stabilization

The LHB is retrieved subcutaneously to the lateral acromion, spanning over the superior ACJ capsule. Using an image intensifier and, if needed, an additional anterolateral portal, the LHB can be anchored to the lateral acromion using a knotless anchor (Figure 5E and F).

#### Wound Closure and Final Imaging

Standard wound closure is performed. Final radiographic images show an anatomic reduction of the ACJ in both vertical and horizontal planes.

#### Rehabilitation

A brace should be worn for the first 6 weeks. The objective is to achieve full shoulder range of motion by 2 months postoperatively. For the first 2 weeks, movement is restricted to the hand and elbow. In weeks 3 and 4, limited passive shoulder motion may begin, with 60° of flexion, 60° of abduction, and 30° of external rotation and internal rotation to the belly. During weeks 5 and 6, external rotation may be increased to 60°, whereas 60° of flexion and 60° of abduction and internal rotation to the belly remain. In weeks 7 and 8, active‐assisted flexion and abduction up to 90° are introduced, along with unrestricted rotational movements. Strengthening exercises may be initiated after 3 months.

## DISCUSSION

Successful reconstruction in the challenging setting of chronic ACJ instability requires restoration of both vertical and horizontal stability while minimizing morbidity and ensuring biological incorporation. Multiple grafts have been proposed, each with inherent advantages and limitations.^3^, ^4^, ^5^, ^6^, ^7^ The modified AutoBAHN technique builds on the principles of the original procedure while addressing specific limitations related to altered anatomy or concomitant pathologies. Instead of preserving the native LHB origin, a free tendon graft can be secured to the scapular neck in different fashions. Onlay and inlay techniques have been evaluated for tenodesis of the short head of the biceps. Similar clinical and functional outcomes have been reported.^22^ Popeye deformity occurred slightly less frequently with an onlay technique (7.8%, n = 17/218) than with inlay technique (11.28%, n = 15/133).^22^ Although not statistically significant, failure rates of the inlay technique were higher (6.86%, n = 7/102) than those of the onlay technique (1.61% n = 2/124).^22^

Biomechanical data show similar load‐to‐failure rates (210 N onlay and 215 N inlay technique).^23^ In the animal model, biomechanical and histological comparisons of the inlay and onlay techniques reveal similar biomechanical attributes, with the onlay technique potentially offering more healing capacities.^24^, ^25^

These techniques come with inherent risks and advantages (Table 2). Potential fracture risk, because of the larger drill hole of the inlay technique, could be increased. Nevertheless, patients may benefit from initial superior stability of the press‐fit fixation. Furthermore, the onlay technique provides advantages in the technical execution because tendon and tension can be adjusted easily. In contrast, handling arthroscopic onlay fixation may require more arthroscopic experience. Pearls and pitfalls are depicted in Table 3. Additionally, the cost of the second anchor must be considered.

**TABLE 2:** Advantages and Disadvantages of the Modified AutoBAHN Technique and of the Different Attachment Possibilities at the Scapular Neck

**Advantages**	**Disadvantages**
•Local, robust, cost‐efficient autograft	•Biomechanical and clinical data have not been published
•Potential superior stability and using only 1 implant when applying the inlay technique	•Inlay technique may increase fracture risk and require more arthroscopic experience for attachment to the scapular neck
•Onlay technique may provide a large tendon‐bone interface and potential superior qualities in tendon‐bone interdigitation while simplifying arthroscopic adjustment of tension and position of the LHB	•Onlay technique may provide potentially inferior stability, and 2 implants are less cost‐efficient

AutoBAHN, autologous biceps acromioclavicular and horizontal neutralization technique; LHB, long head of the biceps tendon.

**TABLE 3:** Surgical Pearls and Pitfalls of the Modified AutoBAHN Technique

**Pearls**	**Pitfalls**
•Carefully free adhesions from LHB before retraction	•Adhesions may lead to LHB tears during retraction
•Use the anteroinferior portal to aid with retraction of the LHB	•Tightly secure the suture limbs of the anchors before drilling the second clavicle canal to prevent entanglement or suture damage
•Create a “parking portal” for tendon retraction and management	
•Use a drill guide for accurate canal placement	•Multiple drill attempts may increase fracture risk
•Mark the tendon with a secure and a running strand for adjustment to the scapular neck	•Trim loose threads of the LHB and attach the sutures in baseball stitch fashion to prevent tear‐outs
•Adjust the image intensifier and use an additional lateral portal for accurate drilling at the lateral acromion	•Ensure secure fixation of the all‐suture anchors before attaching the LHB
•Use the remaining sutures of the pulley system device to achieve 2‐level wound closure at the clavicle	•Use additional sutures or a grasper when using the inlay technique

AutoBAHN, autologous biceps acromioclavicular stabilization and horizontal neutralization technique; LHB, long head of the biceps tendon.

Which patients potentially could benefit from either technique is yet to be evaluated. Based on biomechanical and animal model data, the onlay fixation could be more suitable for patients with high demands, prioritizing early functional stability over potential improved properties in tendon‐bone interdigitation of the inlay technique.

The modified AutoBAHN technique maintains the advantages of the original technique, providing a cost‐efficient autologous graft while combining horizontal and vertical stabilization. This modification provides an alternative for anatomical or pathological variations of the LHB. This altered coracoidal insertion could potentially offer advantages in biomechanical properties and tendon‐bone interdigitation.

## DISCLOSURES

The author (M.S.) declares the following financial interests/personal relationships which may be considered potential competing interests: M.S. is a consultant for Stryker; However, in this technique most Implants are produced by Arthrex (Tenodesis Button, Interference Screw and TightRope System). The other authors (T.G.,E.V.E., L.L., T.L.) declare that they have no known competing financial interests or personal relationships that could have appeared to influence the work reported in this article.
